# The Effect of Liposomal Diallyl Disulfide and Oxaliplatin on Proliferation of Colorectal Cancer Cells: In Vitro and In Silico Analysis

**DOI:** 10.3390/pharmaceutics14020236

**Published:** 2022-01-20

**Authors:** Faris Alrumaihi, Masood Alam Khan, Ali Yousif Babiker, Mohammed Alsaweed, Faizul Azam, Khaled S. Allemailem, Ahmad A. Almatroudi, Syed Rizwan Ahamad, Naif AlSuhaymi, Mahdi H. Alsugoor, Ahmed N. Algefary, Arif Khan

**Affiliations:** 1 Department of Medical Laboratories, College of Applied Medical Sciences, Qassim University, Buraydah 51452, Saudi Arabia; f_alrumaihi@qu.edu.sa (F.A.); ababkr@qu.edu.sa (A.Y.B.); K.allemailem@qu.edu.sa (K.S.A.); aamtrody@qu.edu.sa (A.A.A.); ah.algefary@qu.edu.sa (A.N.A.); 2 Department of Basic Health Sciences, College of Applied Medical Sciences, Qassim University, Buraydah 51452, Saudi Arabia; a_khan@qu.edu.sa; 3 Department of Medical Laboratory Sciences, College of Applied Medical Sciences, Majmaah University, Majmaah 11952, Saudi Arabia; m.alsaweed@mu.edu.sa; 4 Department of Pharmaceutical Chemistry and Pharmacognosy, Unaizah College of Pharmacy, Qassim University, Unaizah 51911, Saudi Arabia; f.azam@qu.edu.sa; 5 Department of Pharmaceutical Chemistry, College of Pharmacy, King Saud University, Riyadh 11451, Saudi Arabia; srahamad@ksu.edu.sa; 6 Department of Emergency Medical Services, Faculty of Health Sciences, AlQunfudah, Umm Al-Qura University, Makkah 21912, Saudi Arabia; nasuhaymi@uqu.edu.sa (N.A.); mhsugoor@uqu.edu.sa (M.H.A.)

**Keywords:** diallyl disulfide liposomes, oxaliplatin stealth liposomes, synergism, colorectal cancer, in silico modeling

## Abstract

Diallyl disulfide (DADS) is one of the main bioactive organosulfur compounds of garlic, and its potential against various cancer models has been demonstrated. The poor solubility of DADS in aqueous solutions limits its uses in clinical application. The present study aimed to develop a novel formulation of DADS to increase its bioavailability and therapeutic potential and evaluate its role in combination with oxaliplatin (OXA) in the colorectal cancer system. We prepared and characterized PEGylated, DADS (DCPDD), and OXA (DCPDO) liposomes. The anticancer potential of these formulations was then evaluated in HCT116 and RKO colon cancer cells by different cellular assays. Further, a molecular docking-based computational analysis was conducted to determine the probable binding interactions of DADS and OXA. The results revealed the size of the DCPDD and DCPDO to be 114.46 nm (95% EE) and 149.45 nm (54% EE), respectively. They increased the sensitivity of the cells and reduced the IC_50_ several folds, while the combinations of them showed a synergistic effect and induced apoptosis by 55% in the cells. The molecular docking data projected several possible targets of DADS and OXA that could be evaluated more precisely by these novel formulations in detail. This study will direct the usage of DCPDD to augment the therapeutic potential of DCPDO against colon cancer in clinical settings.

## 1. Introduction

Colorectal cancer (CRC), which includes colon and rectal cancers, is ranked third after breast and lung cancers, with an occurrence rate of 10%, and a total of 18.1 million new cancer cases were recorded in 2020 worldwide. It is the second leading cause of cancer-related death (9.4%) after lung cancer (18%) among all types of cancer [1]. In Saudi Arabia, CRC is one of the most common types of cancer, amounting to 14.7% of total cancer incidences were registered in 2020. However, its cancer-related death was second, with an estimated mortality rate of 14%, compared with breast cancer, with 14.2% [1].

Evidently, CRC appears only in later stages, which can lead to poor prognosis and make the therapy more difficult [2,3,4]. In clinical settings, the primary treatment for CRC is surgical resection, but subsequently, it requires chemotherapy to avoid the risk of recurrence, as it cannot be eradicated completely [5,6,7]. The therapeutic strategies of CRC, which include radiation, surgery, immunotherapy, and chemotherapy, also have certain limitations due to cancer recurrence, drug resistance, and toxicity [8,9,10,11,12]. The development of these outcomes has been shown to be associated with a single-drug therapeutic approach, while the process of carcinogenesis is commonly linked to multiple signaling pathways [13,14,15,16]. Several studies suggest the use of a combined therapeutic strategy to circumvent the problem associated with monotherapy, as it can modulate various targets by synergistic effect with high therapeutic efficacy [17,18,19,20]. The primary challenge in a combined therapy is to select drugs that have different mechanisms and non-overlapping toxicity.

In recent years, the effectiveness of nutraceuticals has received widespread attention in the fight against cancer. It is evident from several epidemiologic studies that several natural foods included in diets may decrease the risk or delay the progression of various diseases such as cancer, cardiovascular disease, and diabetes. The idea of using natural foods to minimize the risk of various types of cancer dates back many decades. It is believed that 33% of total mortality occurring due to cancer could be prevented by including high amounts of natural foods in the diet. Remarkably, nearly 50% of the drugs made available in the market in the last 30 years were either derived directly from plants or chemically modified [21,22,23,24,25].

Garlic has been used widely as a food flavoring spice and traditional herbal medicine against several diseases including cancer, since ancient times [26]. Evidently, several studies suggested that the presence of organosulfur compounds plays an important role in the pharmacological activities of garlic. The intact garlic bulbs contain allin (S-allylcysteine sulfoxide), which is transformed to allicin (diallyl thioulfonate) upon wounding and then finally converted to oil-soluble polysulfides, mainly as diallyl sulfide (DAS), diallyl disulfide (DADS), and diallyl trisulfide (DATS) [27,28,29,30,31]. Interestingly, the DADS found in 66% of all organosulfur compounds has also shown immense potential in health-promoting activities such as antimicrobial, antioxidants, antidiabetic, and anticancer [32,33,34,35,36,37]. Several studies have reported the efficacy of DADS through multiple signaling pathways that control cell proliferation, apoptosis, and metastasis in different cancer models [38,39,40]. The potential of DADS has been shown in reducing the migration and invasion of human colon cancer 205 cells and MDAMB-231 cells by inhibiting MMPs and TNF-α via NF-κ, PI3K/Akt, and MAPK/ERK signaling pathways [41,42]. The exposure of DADS exhibited the induction of apoptosis through p53-mediated pathways in breast cancer and colorectal cancer cells [39,43,44]. Some studies also revealed the DADS-induced autophagy by inhibiting the phosphorylation of PI3K/Akt/mTOR signaling in RAW264.7, leukemia, and osteosarcoma cells [45,46,47]. However, irrespective of retaining broad therapeutic potential, the usage of DADS is limited due to its insolubility in an aqueous medium. Therefore, the preparation of appropriate formulation of DADS is required to broaden its usage in clinical settings. Some studies reported on DADS-containing delivery systems against fungal disease and breast cancer cells [48,49]. Earlier, we developed liposomal formulations of some of the secondary metabolites such as diallyl sulfide (DAS) and thymoquinone (TQ) against skin papilloma and several infectious diseases [50,51,52].

Many researchers have suggested combinations of liposome-based formulations of different chemotherapeutic agents in varying ratios to augment the therapeutic index of the drugs [53,54,55,56]. Recently, the growth inhibition activity of liposomal formulation of curcumin in combination with oxaliplatin and doxorubicin has also been reported in colon cancer systems [57,58]. As evident from several research studies, sterically stabilized, small nanosized, PEGylated long-circulating liposomes have led to a new era in liposome-based drug delivery systems. It showed retarded clearance by the reticular endothelial system (RES), which subsequently led to prolonged drug circulation. Moreover, the small sizes and prolonged circulation of these liposomes led to enhanced extravasation in various solid tumors as the vascular abnormalities associated with tumor angiogenesis. Nevertheless, the sterically stabilized liposomes release drugs for eventual diffusion into the cancer cells but do not participate in the direct interaction with the cancer cells in vitro or in vivo [59,60,61,62].

The present study aimed to develop and characterize the PEG-coated, long-circulating formulations of DADS and OXA and evaluate their potential alone and/or in combination in CRC systems in vitro, following characterization. We also sought to understand several possible molecular targets of DADS and OXA through in silico modeling methods.

## 2. Methods

### 2.1. Reagents

Distearoyl phosphatidylcholine (DSPC), 1,2-distearoyl-*sn*-glycero-3-phosphatiylethanol-amine-*N*-[methoxy(polyethyleneglycol)-2000] (DSPE-PEG_2000_), cholesterol (Chol), and oxaliplatin were procured from Sigma-Aldrich (St. Louis, MO, USA). The cell cytotoxicity assay, Annexin-V FITC, and cellular ROS assay kits were purchased from Abcam (Cambridge, UK). The running, washing, and storage buffers for the flow cytometry were procured from Miltenyi Biotec, Germany. Dulbecco’s modified Eagle medium (DMEM) and fetal bovine serum (FBS) were purchased from Life Technologies, USA. HCT116 (ECACC 91091005) and RKO (ATCC CRL-2577) were commercially purchased from the European Collection of Cell Cultures (ECACC), Salisbury, UK, and the American Type Culture Collection (ATCC), VA, USA, respectively.

### 2.2. Molecular Docking Studies

Three-dimensional crystal structures of numerous targets known to be involved with anticancer drug development were obtained from the Research Collaboratory for Structural Bioinformatics Protein Data Bank (RCSB PDB, http://www.rcsb.org/pdb/home/home.do) (accessed on 10 September 2021). The details of each target with their PDB IDs and docking-predicted binding energies are presented in the results. Each protein was individually handled in Biovia Discovery Studio Visualizer 2020, to remove co-crystallized water molecules, ligands, and cofactors. Gasteiger charges were added to each receptor in MGLTools 1.5.6 and finally saved in the PDBQT format. Three-dimensional chemical structures of diallyl disulfide and oxaliplatin were obtained from the PubChem database in the SDF format, converted to the PDBQT format after merging all non-polar hydrogens and defining torsion tree and rotatable bonds in MGL Tools 1.5.6. Binding sites in each target were allocated according to the native co-crystallized ligands, and grid maps were generated by using Autogrid 4 program. AutoDock 4.2 was used for molecular docking by opting Lamarckian genetic algorithm methodology and default docking protocol for 10 independent runs [63]. At the end of docking, the best poses were selected from the top models of the ligands in each target by examining their binding energy (Δ*G*_binding_, kcal/mol) and non-bonding interaction profile. Biovia Discovery Studio Visualizer 2020, Chimera 1.15, and PyMol 1.7.4 were used to analyze molecular interactions.

### 2.3. Preparation of DADS- or OXA-Entrapped Liposomes

DADS and OXA encapsulated DSPC/Chol/mPEG-DSPE comprising long-circulating stealth liposomes were prepared individually, as described in our previous studies, with minor modification. Briefly, DSPC: Chol (49:21) mmoles with mPEG-DSPE (5% of total phospholipids) and DADS or OXA (1% of total phospholipids) were prepared by the lipid film method. All ingredients were mixed in a round-bottom flask, and then, the solvents were evaporated to make a thin lipid film using a rotary evaporator in a N_2_ environment. Multilamellar vesicles (MLVs) were prepared by hydrating the lipid film with PBS, followed by sonication, to make ULVs, using a probe sonicator. The suspension of ULVs was then extruded using a handheld extruder at ambient temperature sequentially from 400, 200, and 100 nm decreasing pore-sized polycarbonate membranes with 5–10 cycles for each size of the membrane. The unentrapped DADS or OXA was removed as the supernatant was discarded following centrifugation of DADS or OXA containing liposomes at 30,000 rpm for 30 min.

### 2.4. Characterization of Liposomes

#### 2.4.1. The Size, Zeta (ζ) Potential (mV), Poly Dispersity Index (PDI), and Entrapment Efficiency (EE) of DADS- and OXA-Containing and Empty Liposomes

The entrapment efficiency (EE) of DADS and OXA was determined by taking the absorbance at 330 nm and 250 nm using standard plots of DADS and OXA, respectively. The concentrations of DADS and OXA in liposomes were estimated after the disruption of liposomes with 0.5% Triton X-100. The percentage EE of DADS and OXA were determined using the following formula:

$$\%\;\mathrm{Entrapment}\;\mathrm{Efficiency}\;\left(\mathrm{EE}\right)\mathrm{of}\;\mathrm{the}\;\mathrm{drug}=\frac{\mathrm{Liposome}\;\mathrm{entrapped}\;\mathrm{drug}}{\mathrm{Total}\;\mathrm{drug}}\times 100$$

The mean particle sizes, zeta potentials, and PDI of prepared liposomes were determined by dynamic light scattering (DLS) using the Zetasizer Nano System (Malvern Instruments, Malvern, Worcestershire, UK).

#### 2.4.2. In Vitro Stability of DCPDD and DCPDO and Release Kinetics of DADS and OXA

The stability of DADS-DCPDL and OXA-DCPDL liposomes was determined by in vitro drug release assay at 37 °C, as described earlier [52]. Briefly, 1 mL of DADS or OXA containing prepared liposomes were taken in dialysis bags (MWCO 3.5 kDA) and dialyzed for the next 24 h against 20 mL of PBS, with constant slow stirring. The sample (1mL) was collected at various time points of 0.1, 0.2, 0.4, 0.8, 1, 2, 4, 8, 12, and 24 h, and replaced with 1 mL PBS. The leakage of DADS or OXA was estimated by the formula below after determining the concentration of DADS or OXA by taking the absorbance at 330 nm or 250 nm, respectively, in a UV–Visible spectrophotometer.

The release kinetics of DADS or OXA from the liposomes were assessed by incubating the DADS-DCPDL or OXA-DCPDL in 90% bovine serum. The reaction mixtures were then incubated for 0.1, 0.2, 0.4, 0.8, 1, 2, 4, 8, 12, and 24 h separately, as described earlier [52]. The mixture was then centrifuged immediately after the respective incubation period at 25,000 rpm for 20 min. The leakage and release of DADS or OXA in the PBS and serum, respectively, were estimated by taking the absorbance at 330 nm or 250 nm, correspondingly, in a UV–Visible spectrophotometer by applying the following formula:

$$\mathrm{DADS}\;\mathrm{or}\;\mathrm{OXA}\;\mathrm{release}\;\left(\%\right)=\frac{CnV+\sum_{i=0}^{n}CiVi}{w}\times 100\%$$

where *Cn* is the concentration of DADS or OXA in the solution at the *n* sampling point, and *Ci* is the concentration of DADS or OXA in the solution at the *i* sampling point. *V* is the total solution (20 mL), and *Vi* is the withdrawn volume every time (1 mL).

### 2.5. Cell Cytotoxicity Assay

The percentage of viability at varying concentrations of DADS and OXA were assessed to determine the IC_50_ of free as well as liposomal (DCPDD and DCPDO) formulations in the HCT116 and RKO colon cancer cell lines (Table 1). Briefly, the cells were seeded following 70–80% exponential confluency into 96-wells cell culture plates (10,000 cells/well) for 24 h. The cells were then treated with varying concentrations of DADS, OXA, DCPDD, and DCPDO, as described in Table 1, followed by incubation at 37 °C in a 5% CO_2_ atmosphere for 24, 48, and 72 h. As per the manufacturer’s instructions, the cell cytotoxicity reagent (20 μL) was added after the treatment for the respective period in each well and then incubated at 37 °C, followed by the measurements at 590 nm in a microplate reader. The viability of the cells was measured using the following formula:

$$\%\;\mathrm{Cell}\;\mathrm{Viability}=100\times \frac{\left(A_{sample}-A_{0}\right)}{\left(A_{Ctrl}-A_{0}\right)}$$

where *A_sample_* is the absorbance of treated cells; *A_ctrl_* is the absorbance of untreated cells; *A*_0_ is the absorbance of the background of non-cell control (only media).

The doses of DCPDD and DCPDO were selected after the primary screening of the cells for further analyses as described in Table 2.

### 2.6. Determination of Intracellular ROS Generation by Flow Cytometry

The intracellular ROS levels of living cells were analyzed by the flow cytometry using DCFDA/H2DCFDA-Cellular ROS Assay kit (Abcam, Cambridge, UK), following the manufacturer’s instructions. The cells were divided into seven groups of treatment—namely, G1 (vehicle control of empty liposomes); G2 (+ve control TBHP); G3 (DCPDO IC_10_); G4 (DCPDD IC_10_); G5 (DCPDD IC_25_); G6 (DCPDO IC_10_ + DCPDD IC_10_); G7 (DCPDO IC_10_ + DCPDD IC_25_). Briefly, 2.5 × 10^5^ cells were grown in 6-well plates for 24 h, treated with selected doses, and incubated for 48 h. The cells were harvested, followed by washing in PBS, and incubated with DCFDA (20 μM), for 30 min at 37 °C. The cells were then acquired using MACSQuant Analyzer 10 and analyzed by FlowJo software v10.8.1. The cells from G2 were exposed to 50 μM of ter-butyl hydrogen peroxide (TBHP), 4 h prior to staining with DCFDA.

### 2.7. Annexin V-FITC/PI Apoptosis Assay

The changes in the proportion of cells in the viable, early, and late apoptotic stages were analyzed using Annexin V-FITC/PI Apoptosis Staining Kit (Miltenyi Biotec, Germany). The cells were divided into six groups of treatment—namely, G1 (vehicle control of empty liposomes); G2 (DCPDO IC_10_); G3 (DCPDD IC_10_); G4 (DCPDD IC_25_); G5 (DCPDO IC_10_ + DCPDD IC_10_); G6 (DCPDO IC_10_ + DCPDD IC_25_). The cells were grown, treated, and harvested, as stated earlier. The cells were then incubated with Annexin V-FITC and PI in binding buffer, at room temperature, and for 15 min in the dark, following the manufacturer’s instructions. The samples were acquired using MACSQuant Analyzer 10 and analyzed by FlowJo software v10.8.1.

### 2.8. Statistical Analysis

The mean values and standard errors for all samples were calculated for different treated groups. The significant difference between the groups was measured by the One-way and Two-way ANOVA, as well as Tukey’s multiple comparison tests, using Prism 9. *p*-value < 0.05 was considered statistically significant.

## 3. Results

### 3.1. Molecular Docking Studies

**Figure 1:**
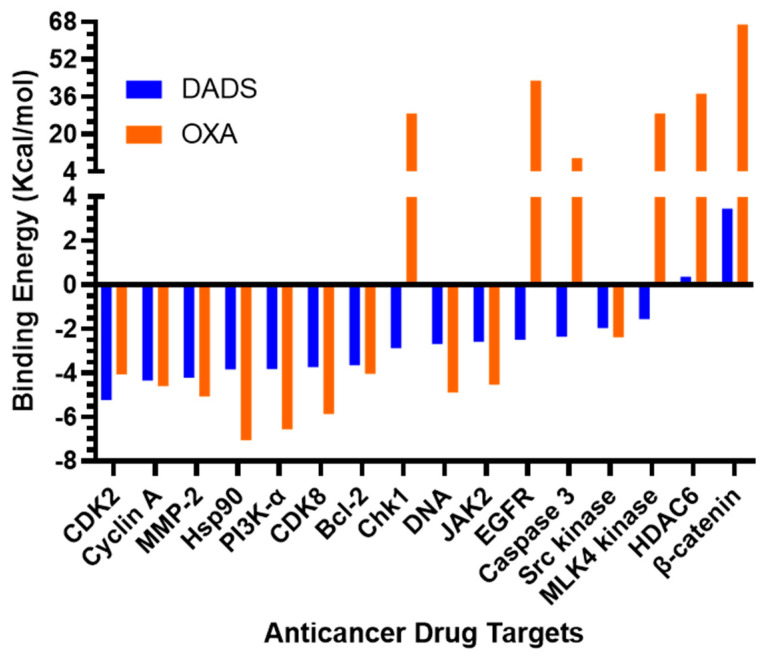
A bar plot of molecular docking-predicted binding energy in kcal/mol and several anticancer drug targets for DADS and OXA.

**Figure 2:**
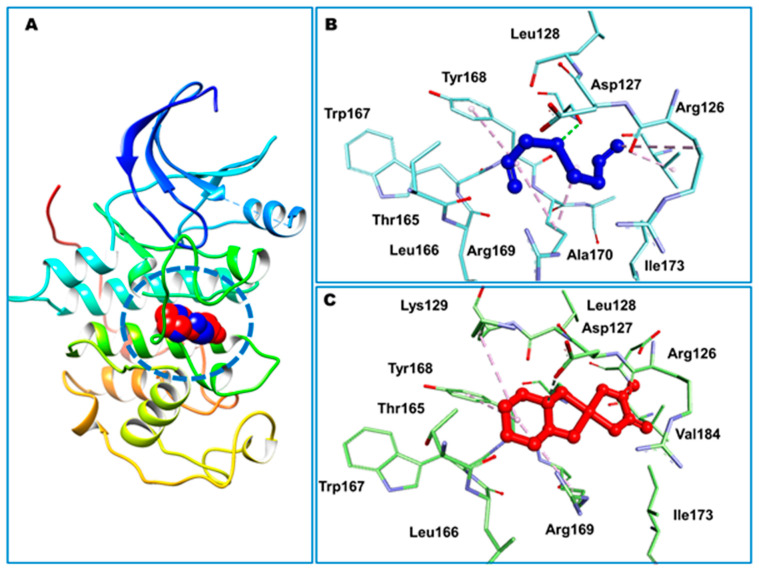
Docked compounds DADS and OXA, shown in blue and red color, respectively. Cyclin-dependent kinase 2 protein is in ribbon style, while docked ligands occupying the binding site are presented as CPK rendering (**A**). Minimum energy conformation of DADS (**B**) and OXA (**C**) are shown as ball and stick, while interacting residues are displayed as sticks. Intermolecular interactions are depicted as broken lines.

**Figure 3:**
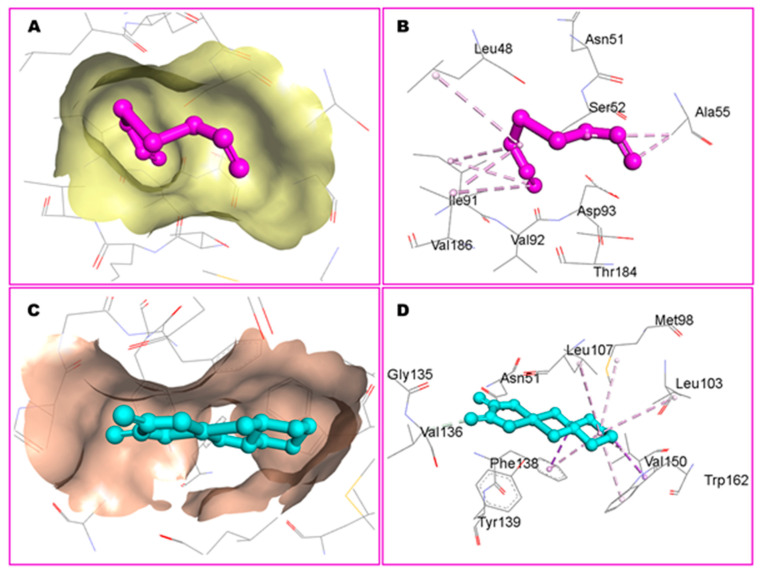
Binding site of Hsp90 protein (shown as surface in **A**,**C**) accommodating docked DADS and OXA, depicted in purple and cyan color, respectively. Residues of the binding site of Hsp90 protein are shown with line styles (**B**,**D**).

The molecular docking data predicted binding affinity in terms of binding energies of DADS and OXA against the DNA and 15 other potential anticancer protein targets (Table 3, Figure 1). As depicted in Table 3 and Figure 1, the DADS exhibited maximum affinity against CDK2, with a minimum binding energy of −5.23 kcal/mol, while OXA displayed −2.11 kcal/mol for this target. However, Hsp90 protein was most favorable for OXA, with −7.06 kcal/mol, but moderate affinity was observed in the case of DADS, showing −3.84 kcal/mol. An intermolecular interaction map of the docked compounds against cdk2 and Hsp90 proteins is demonstrated in Figure 2 and Figure 3, respectively. The hydrophilic and hydrophobic contacts were involved in the intermolecular interactions of both docked compounds against the macromolecular targets used in this study. In particular, the binding cavity of the cdk2 protein is surrounded by residues such as Arg126, Asp127, Leu128, Lys129, Thr165, Leu166, Trp167, Tyr168, Arg169, Ile173, and Val184, providing a profound interaction platform for DADS and OXA compounds (Figure 2 and Figure 3). As depicted in Figure 3, intermolecular contacts highlight the residues of the binding cavity of Hsp90 protein. Asn51 contributed a polar interaction with OXA in terms of hydrogen bond, whereas DADS was devoid of such contacts within the binding site. Amino acid residues involved in non-polar connections include Met98, Leu103, Leu107, Gly135, Val136, Phe138, Tyr139, Val150, and Trp162 (Figure 3).

### 3.2. Characterization of Liposomes

#### 3.2.1. Size, PDI, ζ Potential, and EE

**Figure 4:**
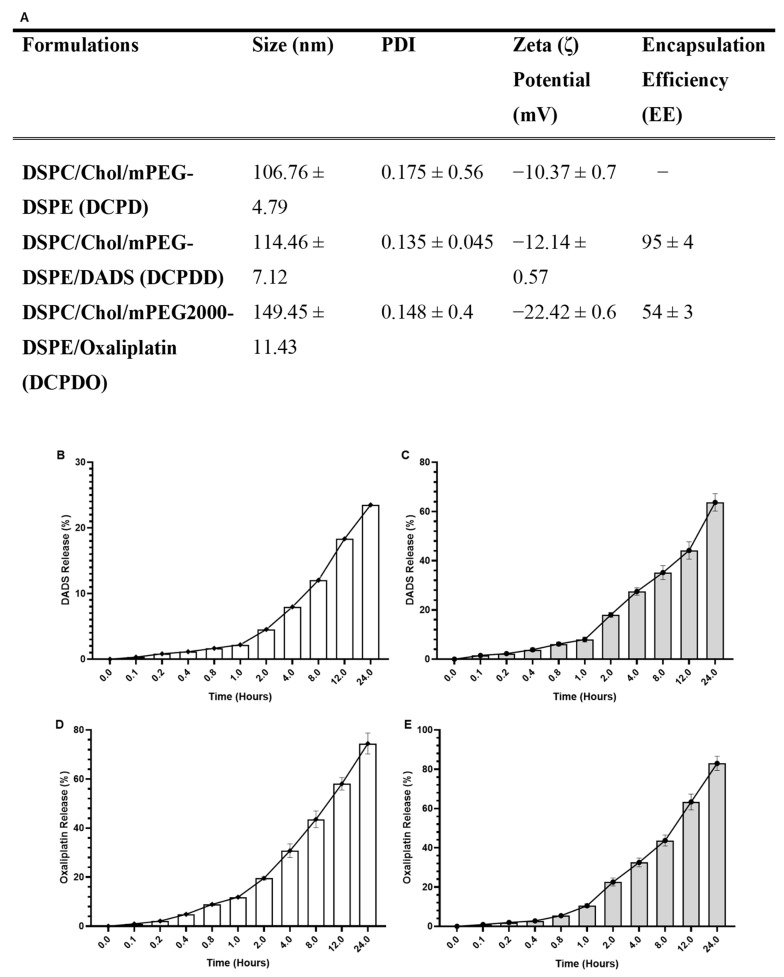
Characterization and in vitro stability and release kinetics of DCPDD and DCPDO: (**A**) size, PDI, zeta potential and entrapment efficiencies; (**B**) stability of DCPDD in PBS; (**C**) release kinetics of DCPDD from the liposomes into the serum; (**D**) stability of DCPDO in PBS; (**E**) release kinetics of DCPDO from the liposomes into the serum The values are expressed as mean ± SE of three independent experiments.

As shown in Figure 4, the DLS data showed the mean particle size of DCPD to be 106.76 nm, while DCPDD and DCPDO were measured to be 114.46 nm and 149.43 nm, respectively, with <0.2 PDI homogeneity in all formulations of liposomes. The zeta potential of the prepared liposomes was measured as −10.37 mv (DCPD), −12.14 mv (DCPDD), and −22 mV (DCPDO), while EE was calculated to be 95% in DCPDD and 54% in DCPDO (Figure 4A).

#### 3.2.2. In Vitro Stability of DCPDD and DCPDO and Release Kinetics of DADS and OXA

The results demonstrated the stability of DCPDD and DCPDO as 23.5% DADS and 74.4% leakage of DADS and OXA were measured, respectively, at 37 °C in the PBS medium (Figure 4B,D). The results from the release kinetics of DADS in the serum revealed that 1.5%, 2.25%, 3.83%, 8.0%, 18%, 27%, 35%, 44.16%, and 63.66% DADS was released after 0.1 h, 0.2 h, 0.4 h, 0.8 h, 1 h, 2 h, 4 h, 8 h, 12 h and 24 h, respectively (Figure 4C). The release kinetics data of DCPDO showed 0.9%, 2.0%, 2.76%, 5.43%, 10.53%, 22.6%, 32.53%, 63.33%, and 83%, correspondingly from 0.1–2 h (Figure 4E).

### 3.3. Effect of DADS and OXA on Cellular Proliferation and IC_10_, IC_25_, IC_35_, and IC_50_ at Varying Doses of DADS, OXA, DCPDD, and DCPDO in HCT116 and RKO Colon Cancer Cell Lines

**Figure 5:**
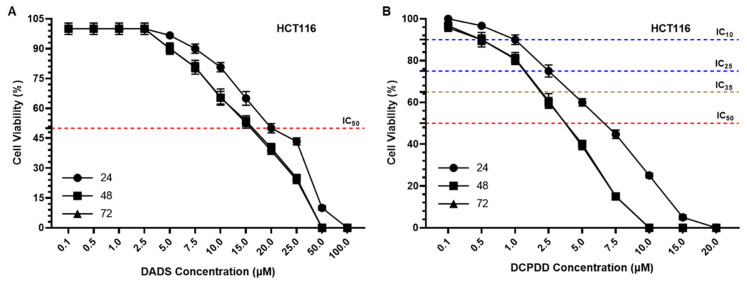

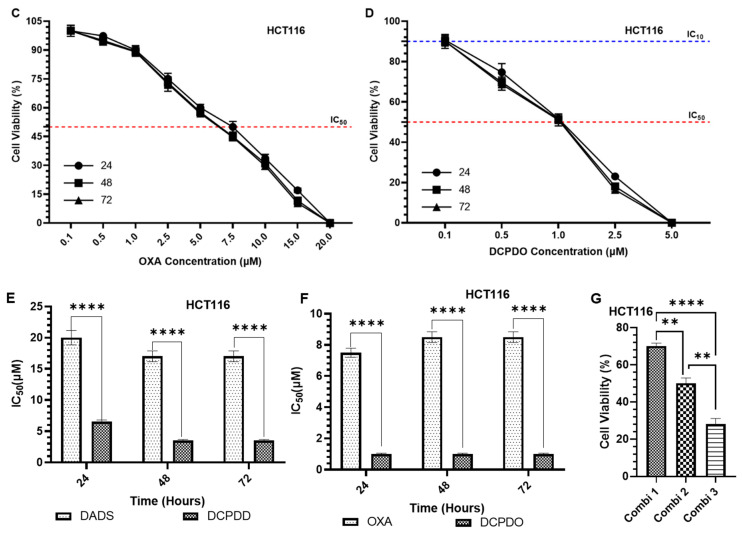
Effect of DADS, DCPDD, OXA, and DCPDO at varying concentrations on cellular proliferation by cell cytotoxicity assay at 24 h, 48 h, and 72 h in HCT116 colon cancer cells (**A**), DADS (**B**), DCPDD, (**C**) OXA, (**D**) DCPDO, (**E**) IC_50_ of DADS and DCPDD, (**F**) IC_50_ of OXA and DCPDO, (**G**) and cell Viability by combinations of DCPDD and DCPDO. The values are expressed as mean ± SE of three independent experiments. ** Significant difference between the treated groups, *p*-value < 0.01, **** Significant difference between the treated groups, *p*-value < 0.0001.

**Figure 6:**
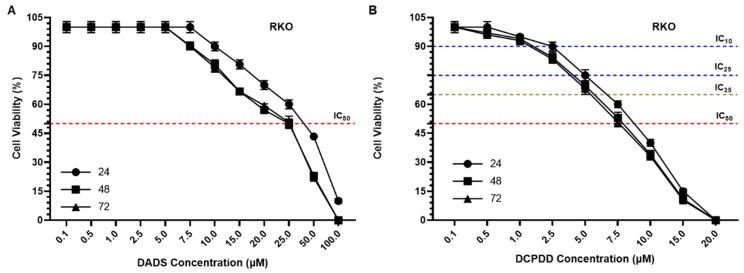

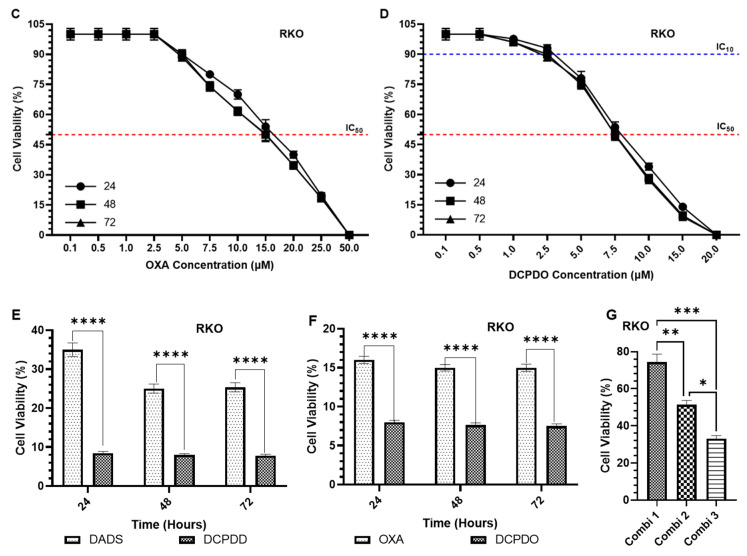
Effect of DADS, DCPDD, OXA, and DCPDO at varying concentrations on cellular proliferation by cell cytotoxicity assay at 24 h, 48 h, and 72 h in HCT116 colon cancer cells (**A**) DADS, (**B**) DCPDD, (**C**) OXA, (**D**) DCPDO, (**E**) IC_50_ of DADS and DCPDD, (**F**) IC_50_ of OXA and DCPDO, and (**G**) cell viability by combinations of DCPDD and DCPDO. The values are expressed as mean ± SE of three independent experiments. * Significant difference between the treated groups, *p*-value < 0.05, ** Significant difference between the treated groups, *p*-value < 0.01, *** Significant difference between the treated groups, *p*-value < 0.001, **** Significant difference between the treated groups, *p*-value < 0.0001.

The results from the cell cytotoxicity assays revealed high sensitivity of DCPDD and DCPDO while comparing DADS and OXA, respectively (Figure 5). According to the data, the IC_50_ of DADS was measured as 20.0 μM after 24 h, while 17.0 μM after 48 h and 72 h in HCT116 cells. However, a several-fold decrease was found in the concentration of DADS by DCPDD, as the IC_50_ was observed to be 6.4 μM (24 h) and 3.5 μM after 48 h and 72 h (Figure 5A,B,E). Noticeably, the treatment period of 48 h and 72 h showed high sensitivity in comparison with 24 h, while no significant difference was found within 48 h and 72 h. Therefore, further study was then conducted for 48 h during which the IC_10_, IC_25_, and IC_35_ of DCPDD were selected to make combinations with the IC_10_ of DCPDO. As depicted in Figure 5G, the cell viability was measured as 70%, 50%, and 28% by combi 1, combi 2, and combi 3, respectively, in HCT116 cells. The results showed that the IC_50_ of free OXA was 7.5 μM (24 h) and 8.5 μM (48 h and 72 h), while it was decreased to only 1 μM after 24, 48, and 72 h (Figure 5C,D,F). The IC_50_ of DADS was estimated as 35.0 μM (24 h), 25.0 μM (48 h), and 25.3 μM (72 h) in RKO cells, while 8.4 μM (24 h), 8.0 μM (48 h), and 7.83 μM (72 h) were recorded by DCPDD (Figure 6A,B,E). In RKO cells, the IC_50_ of OXA was determined as 16 μM (24 h) and 15 μM (48 h and 72 h), whereas it decreased to 8.0 μM (24 h), 7.63 μM (48 h), and 7.5 μM (72 h) by DCPDO (Figure 6C,D,F). Similar to HCT116 cells, the RKO cells were also treated with the selected doses of DCPDD and DCPDO using cell cytotoxicity assay, for 48 h. As shown in Figure 6G, the viability of the cells was estimated as 74.33%, 51.33%, and 33% by combi 1, combi 2, and combi 3, respectively. The combination of DCPDD and DCPDO clearly showed the synergistic potential in very low concentrations, as combi 3 showed more than 50% inhibition of cells, and therefore, combi 1 and combi 2, along with their single doses, were selected further cellular analyses.

### 3.4. Effect of DCPDD and DCPDO on the Intracellular ROS by Flow Cytometry in HCT116 and RKO Colon Cancer Cell Lines

**Figure 7:**
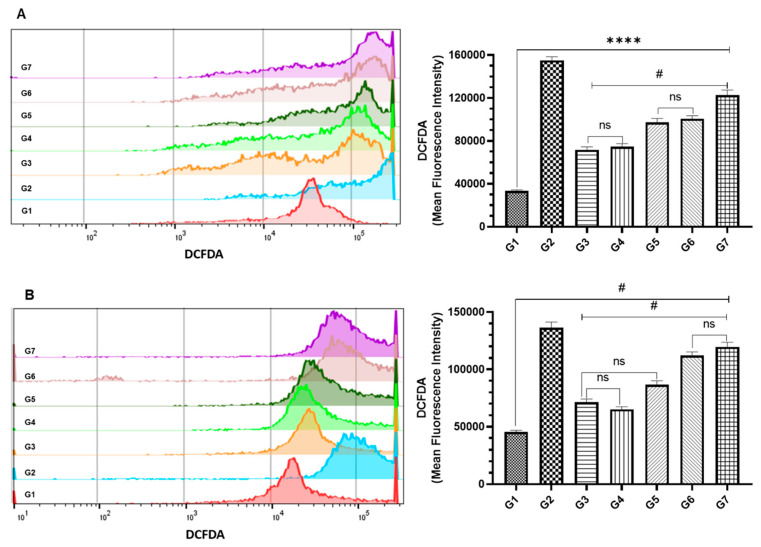
Effect of DCPDD and DCPDO on the induction of cellular ROS by flow cytometry as MFI of DCFDA in colon cancer cells: (**A**) HCT116 cells; (**B**) RKO cells. The doses of DCPDD were selected as IC_10_ and IC_25_, while IC_10_ of DCPDO is summarized in Table 2 in Materials and Methods. The treatment was divided in seven groups—namely, G1 (vehicle control of empty liposomes); G2 (+ve control TBHP); G3 (DCPDO IC_10_); G4 (DCPDD IC_10_); G5 (DCPDD IC_25_); G6 (DCPDO IC_10_ + DCPDD IC_10_); G7 (DCPDO IC_10_ + DCPDD IC_25_). The values are expressed as mean ± SE of three independent experiments. ^ns^ No Significance within the groups, **** Significant difference between the treated groups, *p*-value < 0.0001. ^#^ Significant difference between the treated groups.

As depicted in Figure 4, the combinations of DCPDD and DCPDO showed great potential in both of the colon cancer cells treated with IC_10_ and IC_25_ of DCPDD but kept the dose of DCPDO as IC_10_. The data showed a ~fivefold increase in the induction of ROS by the inducer (G2), as the DCFDA was measured to be 155,132 MFI, while 33,375 MFI was estimated in the vehicle (G1)-treated HCT116 cells (Figure 7A). As shown in Figure 7A, the significant elevation in the ROS generation was also observed in cells treated with IC_10_ of DCPDO (G3), IC_10_ (G4), and IC_25_ (G5) of DCPDD, which were analyzed to be 71,496 MFI (G3), 74,553 MFI (G4), and 97,162 MFI (G5). However, combi 1 (G6) and combi 2 (G7) demonstrated superior efficacy, as 100,571 MFI and 122,717 MFI were analyzed in them, respectively (Figure 7A). Similarly, ROS was increased more than fourfold by G2, as it was measured at 127,800 MFI, compared with 42,675 MFI of G1 (Figure 7B). The data also demonstrated a significant induction in the generation of cellular ROS by G3, G4, and G5, as it was estimated to be 71,263 MFI, 650,567 MFI, and 86,493 MFI, respectively (Figure 7B). However, the combination showed a synergistic effect with great efficacy in G6 and G7, with values of 112,150 MFI and 119,500 MFI. The analysis revealed ~2.5-fold and 2.63-fold increases in ROS generation by G6 and G7, respectively, compared with G1 (Figure 7B).

### 3.5. Effect of DCPDD and DCPDO on the Induction of Apoptosis by Flow Cytometry in HCT116 and RKO Colon Cancer Cell Lines

**Figure 8:**
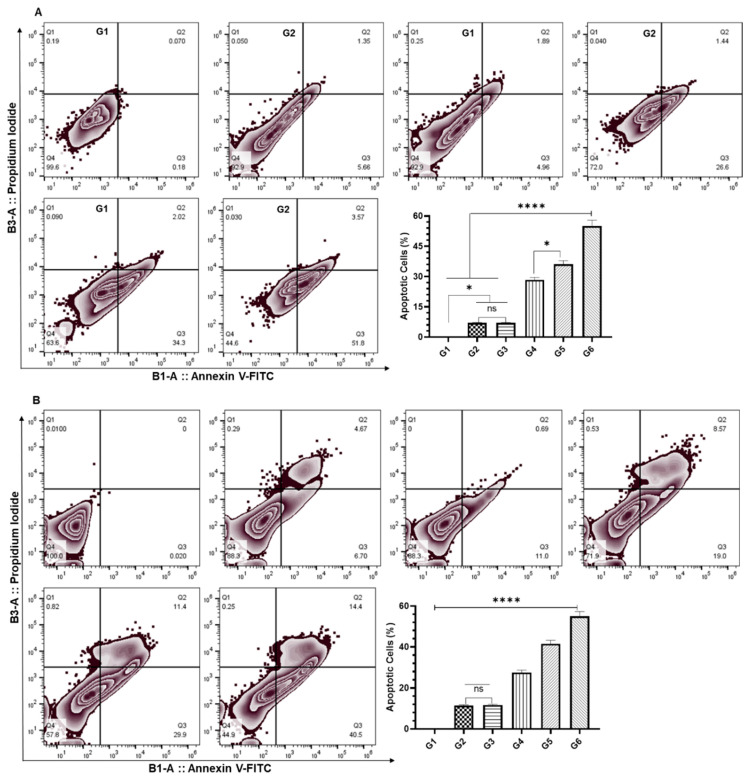
Effect of DCPDD and DCPDO on the induction of apoptosis by flow cytometry in colon cancer cells: (**A**) HCT116 cells; (**B**) RKO cells. The doses of DCPDD were selected as IC_10_ and IC_25_, while IC_10_ of DCPDO is summarized in Table 2 in Materials and Methods. The treatment was divided into six groups—namely, G1 (vehicle control of empty liposomes), G2 (DCPDO IC_10_), G3 (DCPDD IC_10_), G4 (DCPDD IC_25_), G5 (DCPDO IC_10_ + DCPDD IC_10_), and G6 (DCPDO IC_10_ + DCPDD IC_25_). The values are expressed as mean ± SE of three independent experiments. ^ns^ No Significance within the groups, * Significant difference between the treated groups, *p*-value < 0.05, **** Significant difference between the treated groups, *p*-value < 0.0001.

The flow cytometry analysis of colon cancer cells showed that the combi 2 induced apoptosis in 55% of the cells (Figure 8). The data revealed that 7%, 28.4%, 36.17%, and 55% of cells were measured as apoptotic cells in G2 (DCPDO IC_10_), G3 (DCPDD IC_10_), G4 (DCPDD IC_25_), G5 (DCPDD IC_10_ + DCPDO_10_), and G6 (DCPDD IC_25_ + DCPDO_10_), respectively (Figure 8A). Noticeably, early apoptosis was observed in HCT116 colon cancer cells treated with any formulations, as an insignificant number of late apoptotic cells were measured in all treated groups (Figure 8A). The flow cytometry analysis of RKO cells showed 11.37%, 11.7%, 27.5%, 41.5%, and 55% apoptotic cells treated with G2, G3, G4, G5, and G6, respectively. However, the data revealed significant events of late apoptosis in G2 (4.67%), G4 (8.57), G5 (11.4%), and G6 (14.4). Moreover, it was not found in the cells exposed to IC_10_ of DCPDD, clearly pointing to the role of DCPDO in the induction of late apoptosis in combi 1 (Figure 8B).

## 4. Discussion

The current research is the first to establish the liposomal formulation of DADS and summarize the characterization of DCPDD and DCPDO in terms of EE, size, PDI, ζ-potential, stability, and release kinetics in the serum. The lipophilic properties of DADS showed high entrapment efficiency, with 95% in DCPDD, while 56% EE of OXA was found in DCPDO. The rigidity of lipid bilayers makes the liposomes stable, which depends on the choice of lipids and their molar concentration with a high phase-transition temperature (Tc) [32,64,65,66]. The addition of cholesterol with appropriate molar concentration increases the packing of phospholipid molecules and helps to maintain the stability of liposomes. Some researchers demonstrated the high stability of the liposomes using the 2:1 ratio of lipid and cholesterol. The exact ratio of phospholipid and cholesterol has not yet clearly defined for the development of stable and efficient liposomes [67,68,69]. However, our previous studies suggested the high stability of liposomes, comprising DSPC/Chol in the molar ratio of 49:21 [70]. Nevertheless, liposome preparation requires some additional approaches to develop a novel formulation while targeting extra RES to avoid the uptake of liposomes by RES. The coating of liposomes with PEGylated lipids make sterically stabilized liposomes and enables them to retain into the circulation for a longer period and release the drug to the targeted cells [71,72,73]. The present study also characterized the OXA-containing, long-circulating, stealth liposomes, using the same ratio of DSPC:Chol:mPEG as standardized in our lab for PEGylated liposomes [70]. The data showed only 23.5% and 74.5% leakage by DCPDD and DCPDO, respectively. The cumulative release data of stealth liposomes demonstrated 63.66% and 83% release in serums by DCPDD and DCPDO, correspondingly, at 37 °C. Changes in leakage and release kinetics could be seen in DCPDD and DCPDO, due to the hydrophobic and hydrophilic properties of DADS and OXA, respectively, as the DADS is strongly attached to the lipids. Noticeably, no study to date has reported the liposomal formulations of DADS. However, DCPDO also showed increased stability and greater EE of OXA, compared with other studies. Irrespective of the hydrophilic nature of OXA, our DCPDO showed better EE and high stability, with 56% EE, and only 32.5% drug was released in the PBS medium after 4 h, while it was found 83% after 24 h. One of the recent studies showed 25% EE with the release of OXA from PEGylated liposomes, at ~65%, after 4 h [74]. However, another previous study reported more than 90% EE but demonstrated 100% release of OXA within 4 h [75]. Moreover, Lila et al. 2010 reported 22.3% and 18.1% EE in PEGylated cationic and neutral liposomes, respectively (EE) [76]. The stability and EE of DCPDD are attributed to the 49:21 molar ratio DSPC:Chol and the presence of 5% mPEG2000-DSPE of total lipids, which increase the rigidity of the membrane and reduce the permeability, leading to prolonged release of the OXA.

The cellular assays of DCPDD and DCPDO in HCT116 and RKO colon cancer cells demonstrated their high sensitivity, as they reduced the IC_50_ of DADS and OXA several folds. All formulations showed more potential after the incubation of cells for 48 h and 72 h, following treatment of the cells. The synergistic effect of DCPDD was clearly observed in cells exposed to IC_10_ and IC_25_ of DCPDD while keeping the same concentration of DCPDO as IC_10_. The induction of ROS and apoptosis were also measured significantly by DCDO and DCPDD in both of the combinations, confirming our hypothesis. Noticeably, DCPDO showed a significant number of RKO cells in the late apoptotic stage, which was found to continuously increase in combinations. Earlier, some studies reported a significant increase in the late apoptotic stage in HT-29, MCF7S, A549, and Hela cells while treated with OXA [77]. Previously, DADS has been shown to increase the generation of intracellular ROS, resulting in the induction of apoptosis in HCT 116 and HT-29 human colon cancer cells via activation of the p53 signaling pathway [78,79]. The effect of DADS has also been reported to induce ROS in human colon cancer COLO 205 and A549 lung cancer cells through the upregulation of cyclin B, cdc25c-se-216-9, and Wee1 [80,81]. The persistent bioavailability of DADS and OXA in these sterically stabilized liposomes is attributed to apoptosis in the cells. Previous studies showed the effect of OXA-containing gold nanoparticles in HCT116 colon cancer cells, as 7 μM IC_50_ was recorded [82]. The cytotoxic potential of OXA-loaded PEGylated liposomes has been shown by colony-forming assay while treating the cells with more than 20 μM OXA in the liposomes [83]. Furthermore, the IC_50_ of OXA-encapsulating PC, DOTAP, and HSPC had been demonstrated to be 16.5 μM, 9.5 μM, and 15.1 μM in HT-29 colon cancer cells, respectively [84]. Our results clearly indicate an increase in the sensitivity of cells by DCPDO, as only 1 μM and 7.5 μM IC_50_ values were determined in HCT116 and RKO cells, respectively. The results of the present study suggest a detailed exploration of the synergistic effect of DCPDD and DCPDO at very low concentrations in the CRC system. Additionally, the role of projected molecular targets is aimed to be evaluated in vivo, following treatment with DCPDD and DCPDO in CRC animal models.

Molecular docking is a widely used computational technique for the prediction of binding affinities of ligands, as well as the geometry of the ligand–protein complexes [85]. In order to gain insights into the theoretical/hypothetical model for potential interaction modes of DADS and OXA, a molecular docking study was performed against several drug targets related to anticancer drug development. To the best of our knowledge, this is the first study to report the molecular interaction profile of these compounds against various drug targets related to carcinogenesis by molecular docking technique, employing AutoDock 4.2. The docking studies clearly demonstrated that DADS and OXA have suitable molecular frameworks for interaction with various pharmacological targets, which might be attributed to anticancer efficacy in the intrinsic pathway of apoptosis. The variation in the affinity of the docked compounds is obvious because of the differences in their chemical structures. Docking results rendered HDAC6 and beta catenin as the least desirable targets for both compounds, showing binding energy on a positive scale. In general, the compounds displaying positive binding energy are usually weak inhibitors/stimulators of a particular target. Although molecular docking is a robust computational technique for estimating intermolecular interactions between ligand and protein or protein and protein, the technique is incapable of providing a reasonable assessment of the role of the solvation penalty associated with the binding, and hence, further study is warranted to justify the approximation of the binding energy [86].

Notably, CDK2 is the most probable target for eliciting anticancer activity by DADS in docking simulation. There are a number of bioactivities that CDK2 is involved in, such as cell cycle control and DNA replication. CDK2 interacts with and phosphorylates the proteins involved in a variety of biological processes, including intracellular transport, signal transduction, protein degradation, DNA damage, and DNA and RNA metabolism [87]. Similarly, a robust interaction of OXA with Hsp90 is noteworthy because it is an interesting target for cancer therapy due to its significant involvement in oncogenic signaling. When Hsp90 activity is inhibited, multiple signal transduction pathways essential for tumor development and survival are disrupted simultaneously [88]. Recent findings demonstrate that tumor cells use Hsp90 quite differently than normal cells, which explains the drug’s selectivity and implicates a vital role for Hsp90 in carcinogenesis [89]. These findings might serve as starting points for further research into the specific anticancer mechanism of action of the DADS and OXA.

## 5. Conclusions

The current study is the first to develop the DADS-containing, long-circulating, PEGylated stealth liposomes. We found that the stability and efficacy profile of DADS and OXA were augmented in the liposomes developed, with the distinctive ratio of DSPC, cholesterol, and mPEG. We demonstrated the chemosensitizing effect of DCPDD with the lower dose of DCPDO in colorectal cancer cells (CRCs), as they increased apoptosis following the induction of ROS in cancer cells. Further studies are required to understand the molecular mechanism of DCPDD and DCPDO combinations in the treatment of colorectal cancer in vitro and in vivo, focusing on molecular targets of TQ, suggested by molecular docking data.

## Figures and Tables

**Table 1:** The range of the doses of DADS and OXA in free and liposomal formulations for cell cytotoxicity assay in HCT116 and RKO colon cancer cells.

Colon Cancer Cells	Formulations			
	DADS	DCPDD	OXA	DCPDO
HCT116	0.1–100.0 μM	0.1–20 μM	0.1–20 μM	0.1–5 μM
RKO	0.1–100.0 μM	0.1–20 μM	0.1–20 μM	0.1–20 μM

**Table 2:** The selected doses of DCPDD and DCPDO for cellular assays.

Colon Cancer Cells	Formulations			
	DCPDD IC_10_	DCPDD IC_25_	DCPDD IC_35_	DCPDO IC_10_
HCT116	0.5 μM	0.95 μM	2.95 μM	0.1 μM
RKO	1.6 μM	4.0 μM	4.75 μM	2.5 μM

**Table 3:** Molecular docking of DADS and OXA against several anticancer drug targets.

S.N.	Targets	PDB Code	Binding Energy (kcal/mol)	
			DADS	OXA
1	Apoptosis regulator Bcl-2	4LXD	−3.65	−4.04
2	β-catenin	3SL9	3.46	66.91
3	Caspase 3	1RE1	−2.36	9.81
4	Cyclin dependent kinase 2 (CDK2)	2A4L	−5.23	−2.11
5	Cyclin dependent kinase 8 (CDK8)	3RGF	−3.73	−5.87
6	Checkpoint Kinase 1 (Chk1)	2R0U	−2.87	28.89
7	Cyclin A	6GUE	−4.34	−4.6
8	Double-stranded DNA	1AIO	−2.68	−4.89
9	Epidermal growth factor receptor (EGFR)	1M17	−2.5	42.87
10	Histone deacetylase 6 (HDAC6)	5WGI	0.37	37.34
11	Heat shock protein 90 (Hsp90)	4BQG	−3.84	−7.06
12	Janus kinase 2 (JAK2)	3KCK	−2.58	−4.54
13	Mixed-lineage kinase (MLK4)	4UYA	−1.56	28.89
14	Matrix metalloproteinase-2 (MMP-2)	1HOV	−4.22	−5.07
15	Phosphatidylinositol-3 kinase alpha (PI3K-α)	3ZIM	−3.83	−6.56
16	Src kinase	2BDF	−1.96	−2.38

## Data Availability

Not applicable.
